# The RNA editing enzyme APOBEC1 induces somatic mutations and a compatible mutational signature is present in esophageal adenocarcinomas

**DOI:** 10.1186/s13059-014-0417-z

**Published:** 2014-07-31

**Authors:** Giulia Saraconi, Francesco Severi, Cesare Sala, Giorgio Mattiuz, Silvestro G Conticello

**Affiliations:** Core Research Laboratory, Istituto Toscano Tumori, viale Pieraccini 6, 50139 Firenze, Italy

## Abstract

**Background:**

The AID/APOBECs are deaminases that act on cytosines in a diverse set of pathways and some of them have been linked to the onset of genetic alterations in cancer. Among them, APOBEC1 is the only family member to physiologically target RNA, as the catalytic subunit in the Apolipoprotein B mRNA editing complex. APOBEC1 has been linked to cancer development in mice but its oncogenic mechanisms are not yet well understood.

**Results:**

We analyze whether expression of APOBEC1 induces a mutator phenotype in vertebrate cells, likely through direct targeting of genomic DNA. We show its ability to increase the inactivation of a stably inserted reporter gene in a chicken cell line that lacks any other AID/APOBEC proteins, and to increase the number of imatinib-resistant clones in a human cellular model for chronic myeloid leukemia through induction of mutations in the *BCR-ABL1* fusion gene. Moreover, we find the presence of an AID/APOBEC mutational signature in esophageal adenocarcinomas, a type of tumor where APOBEC1 is expressed, that mimics the one preferred by APOBEC1 *in vitro*.

**Conclusions:**

Our findings suggest that the ability of APOBEC1 to trigger genetic alterations represents a major layer in its oncogenic potential. Such APOBEC1-induced mutator phenotypes could play a role in the onset of esophageal adenocarcinomas. APOBEC1 could be involved in cancer promotion at the very early stages of carcinogenesis, as it is highly expressed in Barrett's esophagus, a condition often associated with esophageal adenocarcinoma.

**Electronic supplementary material:**

The online version of this article (doi:10.1186/s13059-014-0417-z) contains supplementary material, which is available to authorized users.

## Background

APOBEC1 (Apolipoprotein B mRNA editing enzyme, catalytic polypeptide 1) is part of the RNA editing complex that physiologically deaminates C6666 to U in the transcript of human Apolipoprotein B, a major component in lipid transport [1-3]. APOBEC1 exerts this function in the small intestine in humans, and in the liver in rodents.

APOBEC1 was the first identified member of the AID/APOBEC protein family, a group of cytosine deaminases that target nucleic acids in a diverse set of pathways to induce C > U changes [4,5]. Most of these proteins are DNA mutators: AID (Activation Induced Deaminase) is essential for all secondary antibody diversification processes [6,7], and the APOBEC3s act in a defense pathway against retroviruses and mobile elements [8,9].

The only well-characterized target for APOBEC1 is the mRNA for Apolipoprotein B; however, additional target mRNAs have been identified [10-12], and it has been also suggested that APOBEC1 regulates mRNA stability through its ability to bind RNA [13,14]. On the other hand, APOBEC1 can also target DNA in bacteria and *in vitro* [15-17]. Based on this activity, other roles have been suggested, from controlling DNA methylation [18,19], to being part of a restriction pathway against retroviruses and mobile elements, similar to the APOBEC3s [20-25].

APOBEC1 expression has been linked to cancer: transgenic mice and rabbits constitutively expressing APOBEC1 in the liver develop hepatocellular carcinoma [26], and APOBEC1 deficiency in cancer-prone APC^min^ mice reduces the number of polyps and tumors in the gastrointestinal tract [27]. The oncogenic potential of APOBEC1 has been attributed mostly to its ability to target RNA [3]. However, we hypothesize that the oncogenic role of APOBEC1 is related to its ability to target DNA. This hypothesis is corroborated by the evidence that aberrant activity of other AID/APOBECs underlies the onset of genetic alterations in human cancer [28-35].

In direct support of this notion, here we show in two cellular models that expression of APOBEC1 induces a mutator phenotype. In addition, we show the presence of the mutational signature of the AID/APOBECs in human esophageal adenocarcinomas, a type of tumor in which APOBEC1 is highly expressed.

## Results and discussion

### Rat APOBEC1 induces a mutator phenotype in a chicken cell line

**Figure 1:**
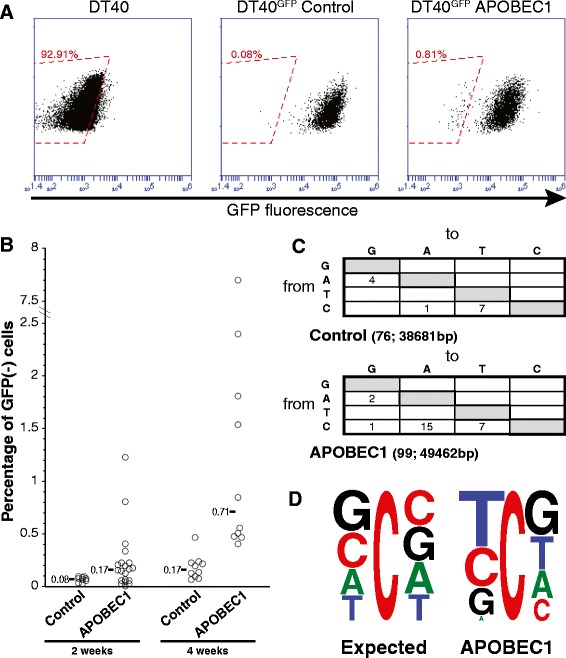
**APOBEC1 induces mutations in DT40 cells. (A)** The inactivation of the EGFP transgene in DT40 cells was assayed by monitoring the size of a distinct EGFP(−) population in transfectants expressing either APOBEC1 or a control plasmid (Figure S1A in Additional file 1). Flow cytometric analysis of representative DT40^GFP^ clones stably transfected with APOBEC1 or with the control plasmid. The red boxed area indicates the EGFP(−) population considered for the analysis. **(B)** Plot depicting the inactivation of the EGFP in independent transfectants, with the median for each construct indicated. The measurements were taken at the indicated times (*P* < 0.05 for each time-point). The sorted EGFP(−) cells did not regain fluorescence after expansion. **(C)** Mutation pattern obtained after sequence analysis of independent EGFP fragments from sorted EGFP(−) APOBEC1- and vector-transfected DT40 cells. The number of sequences analyzed and the total number of bases is indicated in parentheses (*P* = 0.012 by Fisher’s exact test). Only non-clonal mutations were considered, which is likely to underestimate the number of mutations in the case of preferred mutational hotspots. **(D)** Local sequence context for the cytosine residues present on both strands of the analyzed GFP fragments (expected) and for the mutated cytosines (observed) in the APOBEC1-expressing clones.

In order to investigate the ability of rat APOBEC1 - the best characterized among the mammalian homologues - to induce a mutator phenotype, we used DT40 cells, a chicken lymphoma B cell line that has been extensively employed to study the mutational activity of AID [36], the only catalytically active member of the AID/APOBEC family present in the chicken genome [17,37]. A derivative DT40 clone in which AID has been knocked out (sIgM^+^ ψV^−^ AID^−/−^) [38] has enabled us to assay the effects of APOBEC1 in the absence of other DNA mutators. We first inserted a single-copy enhanced green fluorescent protein (EGFP) reporter gene in the DT40 genome, so that inactivation of the EGFP could be easily detected through fluorescence-activated cell sorting (FACS) analysis, in a similar way to that used to study AID-dependent mutations [36]. We then transfected independent DT40^GFP^ clones with constructs expressing either rat APOBEC1 or a control plasmid, and we followed the expression of the EGFP reporter gene in clones selected to stably express APOBEC1 (Figure S1A in Additional file 1). Starting from 2 weeks after transfection we observed the rise of a distinct EGFP(−) population in the clones expressing APOBEC1 (Figure 1A,B). At 4 weeks after transfection, the median percentage of the EGFP(−) cells in the APOBEC1-expressing clones was four times larger than in the control clones. Indeed, sorted EGFP(−) cells remained negative after expansion. It seemed unlikely that this finding could result from an effect of APOBEC1 on RNA, since this would likely alter the overall level of EGFP RNA rather than abolish its expression in individual cells. To confirm this we sorted the GFP(−) population from APOBEC1- and control-transfected cells and sequenced the EGFP coding region to detect the presence of mutations. Approximately a quarter of the sequences bore mutations. The mutation pattern observed in the samples expressing APOBEC1 was different from the mutational background in the controls (Figure 1C): C/G base pairs were preferentially mutated in the APOBEC1 samples, and the changes were mainly transversions, a pattern reminiscent of the one observed after AID-dependent deamination in DT40 cells [38].

Each member of the AID/APOBEC family preferentially targets cytosines within a specific sequence context: this preference can be quite strict (for example, CCC for APOBEC3G, TC for APOBEC3B) or more relaxed (for example, WRC for AID, YC for APOBEC1). Indeed, the sequence context of the mutations at cytosine residues observed in the EGFP reflects that observed *in vitro* and in bacteria for APOBEC1 [15,16,39], especially with regard to the preference for a thymine directly upstream to the mutated C and the avoidance of adenines (Figure 1D; Additional file 2).

### Human APOBEC1 is able to mutate DNA in bacteria

**Figure 2:**
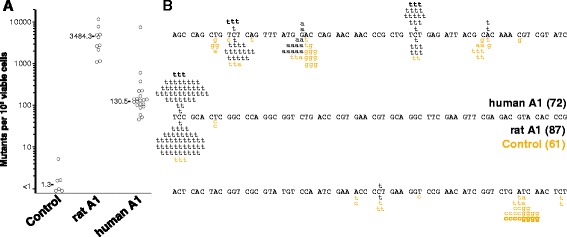
**Human APOBEC1 induces a mutator phenotype in bacteria. (A)** Frequency of rifampicin-resistant clones in cultures of *Escherichia coli* transformed with APOBEC1 expression constructs (rat or human) or empty vector. Each point represents the mutation frequency of an independent overnight culture. The median mutation frequency is indicated. **(B)** Distribution of independent rpoB point mutations identified in rifampicin-resistant clones from bacteria transformed with human APOBEC1 (black changes above the sequence), rat APOBEC1 (black changes below the sequence), or empty plasmid (brown changes below the sequence). The number of independent colonies is indicated in parentheses.

Human APOBEC1 - in contrast to other mammalian homologues - exerts only a minor effect on retroviruses and mobile elements [20,22,24,40]. This has cast doubts on its ability to target DNA. We therefore cloned human APOBEC1 in a bacterial expression vector and used this in the rifampicin-resistance reversion assay, a test widely used to study AID/APOBEC-mediated induction of a mutator phenotype. The outcome of the experiments reveals that human APOBEC1 is effective as a DNA mutator (Figure 2A). Sequence analysis of the *rpoB* gene from rifampicin-resistant clones shows that the mutations induced by human APOBEC1 closely resemble those induced by the rat homologue (Figure 2B).

### APOBEC1 induces a mutator phenotype in human K562 cells

**Figure 3:**
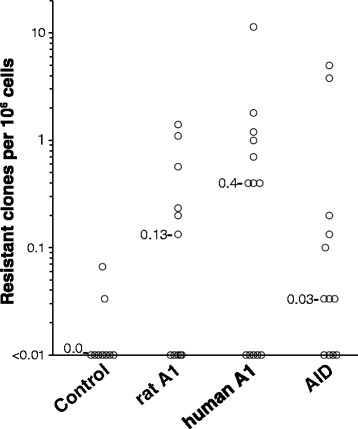
**APOBEC1 induces a mutator phenotype in a human chronic myeloid leukemia cell line.** Frequency of imatinib-resistant clones arising from 10^7^ K562 cells expressing either mammalian APOBEC1s, AID, or a control plasmid seeded in the presence of 1 μM imatinib (control/APOBEC1, *P* < 0.05). Imatinib resistance was confirmed by growing the clones in imatinib-supplemented medium.

Having confirmed that both rat and human APOBEC1 could effectively target DNA, we tested whether the APOBEC1-induced mutator phenotype could affect drug-resistance dynamics in a cancer cellular model. To this end, we used human K562 cells, a model for chronic myeloid leukemia (CML) bearing a *BCR-ABL1* chromosomal translocation. The *BCR-ABL1* fusion gene provides support for the cellular growth, and treatment with imatinib, a tyrosine kinase inhibitor, results in cell death due to blockade of the BCR-ABL1 kinase domain. Mutations in *BCR-ABL1* can produce imatinib-resistant clones: it has been shown that exogenous overexpression of AID induces mutations that can confer resistance to the drug by disrupting the interaction of BCR-ABL1 with the drug [41,42]. AID/APOBECs do not seem to be expressed in these cells, either treated or not with imatinib. After obtaining stably transfected clones, we cultured K562 cells expressing APOBEC1, AID as a positive control, or a control plasmid (Figure S1B in Additional file 1) in the presence of imatinib to select clones resistant to the drug. In line with our expectations, a higher number of colonies was present in the samples expressing AID or the APOBEC1, both rat or human, compared with controls, with APOBEC1-expressing clones outnumbering those expressing AID (Figure 3). After confirming that the selected clones were able to grow in imatinib-supplemented medium, we amplified the region of the *BCR-ABL1* gene encoding the imatinib binding site to evaluate the presence of mutations. Sequence analysis of individual clones confirmed the presence of mutations in the ABL1 tyrosine kinase domain, mostly located at residues near the catalytic pocket (Table 1). Taking into account the number of observed mutations and the fact that the selected phenotype depends on mutations at specific sites of the fusion gene (as opposed to loss-of-function mutations in the EGFP), the type and the sequence context of the mutations observed are not indicative of the AID/APOBEC1 mutational preferences. This is in line with previous studies in which A/T mutations represented 33 to 50% of all unique *BCR-ABL1* mutations induced by AID [41,42].

**Table 1:** **List of the non-clonal mutations identified in the** ***BCR-ABL1*** **fusion gene from imatinib-resistant K562 clones**

**Samples**	**Position**	**AA change**	**Codon change**	**Sequence context**
	1,503	E495G	G**A**A > G**G**A	
Control	1,472	R491Q	C**G**G > C**A**G	GC**C**GG
	1,503	Silent	TT**T** > TT**C**	
	633	G100C	**G**GC > **T**GC	GC**C**TA
AID	395	Silent	AA**A** > AA**G**	
	568	G190D	G**G**C > G**A**C	TG**C**CA
	633	T212A	**A**CG > **G**CG	
	987	G155D	G**G**C > G**A**C	GG**C**CA
	607	L203M	**C**TG > **A**TG	CC**C**TG
Rat APOBEC1	613	E205K	**G**AG > **A**AG	CT**C**GG
	764	K255T	A**A**G > A**C**G	
	987	Silent	GG**G** > GG**T**	TT**C**CC
	1,245	silent	AG**C** > AG**T**	AG**C**CG
	758	R253H	C**G**C > C**A**C	TG**C**GC
	841*	silent	**C**TG > **T**TG	AG**C**TG
	697	H233D	**C**AT > **G**AT	TT**C**AT
Human APOBEC1	699	H233D	CA**T** > GA**C**	
	1,149*	Silent	GC**C** > GC**A**	GC**C**AT
	1,245*	F415L	TT**T** > TT**G**	

The region analyzed (encompassing exon 13 of BCR and exon 9 of ABL1) includes the imatinib-binding region of the fusion gene. The asterisk indicates mutations found in the same clone. The local sequence context for the mutations at cytosines is shown. Compared to the AID-induced mutations found in previous reports (mutations in approximately 30% of the sequences) [41,42], we found approximately one mutation in each of the clones analyzed. This is explained by the different procedures we used to select resistant clones: whereas the other studies focused on competing bulk populations of AID-transfected GFP(+) cells and control GFP(−) cells, we analyzed individual clones arising from the same number of cells plated in the presence of imatinib.

### APOBEC1 expression in esophageal adenocarcinomas

Thus, our findings show that APOBEC1 can induce somatic mutations in vertebrate cells. Such an APOBEC1-induced mutator phenotype could play a role in the onset of cancer previously observed in mice. On the other hand, there is no evidence so far linking APOBEC1 to any human cancer.

**Figure 4:**
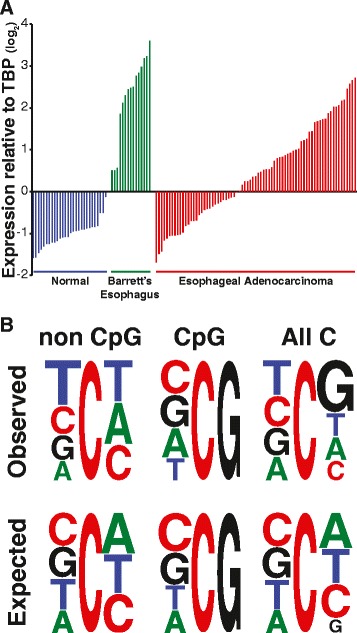
**Upregulation of APOBEC1 and mutational context in esophageal adenocarcinomas. (A)** Expression levels of APOBEC1 in a cohort of samples from normal esophagus, Barrett’s esophagus and EACs. The data were extracted from a recent study analyzing the transcriptome of EAC using microarrays [44], normalized on TATA-binding protein (TBP) expression levels and shown as median-centered Log2 values. Differences between the various sets of data are statistically significant (*P* < 10^−4^ by one-way ANOVA coupled with Tukey’s test). Details of APOBEC1 expression patterns from this and other studies are shown in Additional file 3. **(B)** Sequence context for the mutated cytosine residues in EAC exomes from a recent study [47]. The trinucleotide sequence context is shown for mutations at Cs (All C), at CpG and at non-CpG sites as a weblogo [48]. The expected sequence context for random mutations at cytosines in the human exomes is shown (*P* < 0.0001 for the first position at non-CpG sites by Chi-square analysis).

We therefore searched the Gene Expression Omnibus (GEO) repository [43] for specific types of cancer in which APOBEC1 was overexpressed. We found that, in striking contrast to normal esophageal mucosa, the expression levels of APOBEC1 in esophageal adenocarcinomas (EACs) are comparable to those observed in the small intestine, where APOBEC1 is expressed at its highest levels [44-46] (Figure 4A; Additional file 3). Indeed, in individual matched pairs of normal esophageal mucosa and EAC, APOBEC1 is consistently overexpressed in EACs (Figure S3B in Additional file 3). In comparison, other AID/APOBECs such as AID and APOBEC2 are not expressed. An increased expression of APOBEC3s in EACs cannot be excluded: in the dataset from Kim *et al*. [44] there is a statistically significant increase of APOBEC3A (Additional file 3), which is lost when comparing normal/EAC pairs (data not shown); on the other hand, in the dataset from Kimchi *et al*. [45], it is APOBEC3B that is increased in EACs, albeit not at the levels of APOBEC1 (data not shown).

The assessment of the expression levels of the APOBEC3s in the datasets from Kimchi *et al.* [45] and Stairs *et al.* [46] is confused by the U133A Affymetrics platform used (due to cross-hybridizing probes, as discussed in Burns *et al.* [30]). On the other hand, the data from Kim *et al.* [44] could be informative of the expression levels of several APOBEC3s (see discussion in Additional file 4).

Interestingly, a marked increase of APOBEC1 is also found in Barrett’s esophagus, a condition closely related to adenocarcinomas, both epidemiologically and genetically, as the same hallmark DNA/genomic alterations are seen in both [49-52]. In contrast, this is not the case for the other AID/APOBECs (Figure S3A in Additional file 3). The overexpression of APOBEC1 is in line with the metaplasia that is characteristic of Barrett’s esophagus, whereby the normal esophageal epithelium is replaced by cells with intestinal features.

### AID/APOBEC mutational signature in esophageal adenocarcinomas

In human tumors derived from mature B cells there are characteristic chromosomal translocations and other mutations that can be regarded as a veritable AID mutational signature [28,53]. Other AID/APOBECs have been shown to induce DNA damage and somatic mutations [30,54,55], and they are involved in kataegis, clusters of mutations observed in some cancer genomes [29,32,35,56,57]. An AID/APOBEC mutational signature can be observed in genes highly mutated in cancer [39] and in cancer genomes and exomes [31,33-35,58]. Based on this, we have looked at the data from a recent study on EACs [47] in which at least two mutational signatures had been observed: A to C transversions in the context of the ApA dinucleotide, and C to T transitions at CpG sites. Our re-analysis shows that mutations at non-CpG sites represent about 55% of all mutations at Cs, and the sequence context observed in these non-CpG mutations is similar to that ascribed to AID/APOBEC action (Figure 4B). Such sequence context also resembles that observed for APOBEC1 [15,16,39] as well that we found in APOBEC1-expressing DT40 cells (Figure 1D).

The mutational sequence context at position −1 in EACs is closer to that observed in tumor types in which APOBEC3B is expressed alongside other AID/APOBECs, including APOBEC1 [31]. Moreover, an analysis of the dinucleotides 5’ to a mutated C shows that the ratio between pyrimidine and purine residues at position −2 varies among the tumor types in which an AID/APOBEC mutational signature is present [30,31,33-35,58]. Indeed, the pyrimidine/purine ratio in EACs is the lowest among the different tumors, with the tumors with high expression of APOBEC3B displaying the highest ratio (Additional file 5).

There is no direct evidence of correlation between mutation signature and APOBEC1 expression because of the lack of expression data in Dulak *et al.* [47]. However, different studies show variable levels of APOBEC1 overexpression in EACs (Figure 4; Figure S3A-C in Additional file 3), providing indirect evidence of such correlation. It is noteworthy that APOBEC1 is markedly overexpressed in all samples from Barrett’s esophagus (Figure 4; Figure S3A,C,D in Additional file 3).

Indeed, a recent analysis of the somatic mutations in Barrett’s esophagus reports that 71% of all mutations are targeted to C/G base pairs, with a strong bias towards transition mutations (Ts/Tv ratio at CpH sites of 1.69) [52]. Such a trend is present also in EACs associated with Barrett’s esophagus compared with those that are not associated (Additional file 6; Ts/Tv of 1.22 versus 1.01). If we consider the marked overexpression of APOBEC1 in Barrett’s esophagus, it is suggestive that the AID/APOBEC mutational signature is more evident (albeit not significantly different) in EACs associated with Barrett’s esophagus compared with those that are not (Additional file 7).

## Conclusions

DNA deaminases perform various functions within an organism. Unlike the APOBEC3 genes, which physiologically exhibit a broad expression pattern [59], the expression of AID and of APOBEC1 is limited to few tissues in which they exert their physiologic activity. Ectopic expression of AID (especially outside the B-cell lineage) results in mutations and cancer formation [60,61]. This suggests that tissues and cells that require these deaminases in the nucleus and hence near their own genome have developed protective regulatory mechanisms. This is seen in mature B cells, where AID expression is regulated at the transcriptional level as well as in terms of protein stability and localization. On the other hand, cells that do not physiologically express these deaminases could lack this layer of protection and be more susceptible to self-induced DNA damage (for example, [60,62]).

By analogy, we can presume that cells physiologically expressing APOBEC1 have devices to protect them from its aberrant activity, whereas those not normally expressing APOBEC1, such as the esophageal epithelium, may have no such protection. We have shown here that APOBEC1 can induce mutations in genomic DNA. Together with previous findings linking APOBEC1 presence/absence with the induction of tumors in mice [26,27], our experimental results and our observations in EACs indicate that APOBEC1 could be involved in the onset of cancer by targeting genomic DNA directly.

We do not know yet at which stage in EAC oncogenesis AID/APOBECs exert their mutagenic potential; however, since Barrett’s esophagus can be regarded as a precursor of EAC, and since genomic alterations of EAC are already seen in Barrett’s esophagus [49-52], we surmise that AID/APOBECs play a role at an early stage, even though further studies will be needed to assess the specific contribution of APOBEC1 compared with other AID/APOBECs. In addition, we must consider that, through RNA targeting, APOBEC1 can alter a number of cellular functions [11,13,14,27]. It is possible, therefore, that these changes, associated with APOBEC1 mutagenic activity, may also be boosting its oncogenic potential and drive both the onset and the progression of cancer.

## Materials and methods

### Plasmids and mutator assay in bacteria

The EGFP-expressing construct used for the DT40 experiments was built by subcloning on a pBluescript SK + backbone the beta-actin promoter (XhoI/NheI), the EGFP coding sequence (NheI/KpnI) and a Blasticidin-S resistance cassette (BamHI). Rat and human APOBEC1 were PCR-amplified (rat forward primer AAAGCTAGCATGAGTTCCGAGACAGGCCCTGTA, rat reverse primer AAATGTACAAGATCTCATTTCAACCCTGTGGC; human forward primer AAAGCTAGCATGACTTCTGAGAAAGGTCCT, human reverse primer AAATGTACAAGATCTCATCTCCAAGCCACAGAAGG) and cloned into the pAIDexpressPuro2 expression vector [63] under the control of the beta-actin promoter. Depending on the restriction enzymes used for cloning, the final constructs encoded either APOBEC1 or APOBEC1-IRES-EGFP (NheI-BsrGI or NheI-BglII). The construction of the AID-IRES-EGFP expression construct is detailed in [64]. An empty plasmid with a puromycin cassette [65] was used as control for the DT40 experiments. An analogous one expressing EGFP was used in the experiments on K562 cells. The rat APOBEC1 vector for bacterial expression was described in Harris *et al.* [15]. The human APOBEC1 coding sequence was cloned into pTrc99a (forward primer, TTTCCATGGCCATGACTTCTGAGAAAGGTCC; reverse primer, AAATGTACAAGATCTCATCTCCAAGCCACAGAAGG; NcoI/BglII). The rifampicin-resistance reversion assay used to test the induction of a mutator phenotype was performed as described in Petersen-Mahrt *et al.* [66] and Harris *et al.* [15], but with induction of the AID/APOBEC at 18°C for 24 hours in order to increase the viability of the bacteria and to obtain better resolution (Additional file 8).

### Cells, transfections, and protein expression

sIgM^+^ ψV^−^ AID^−/−^ DT40 cells [38] were maintained in RPMI1640 9% fetal bovine serum (FBS), 1% chicken serum (Life Technologies, Carlsbad, CA, USA), 50 μM 2-mercaptoethanol at 37°C in 5% CO_2_, and transfected as previously described [63]. Cells were selected with 25 μg/ml blasticidin or 0.25 μg/ml puromycin, depending on the plasmid used. DT40^GFP^ cells were prepared by selecting independent clones stably transfected with the EGFP construct. Southern blot analysis using an EGFP fragment as probe were performed to select for DT40^GFP^ clones bearing a single EGFP copy.

K562 cells were maintained in DMEM supplemented with 10% FBS at 37°C in 5% CO_2_; 10^6^ cells and 5 μg of plasmid were used to electroporate K562 cells (250 V, 950 μF, Biorad GenePulserII Hercules, CA, USA). K562 cells were selected with 3 μg/ml puromycin.

EGFP fluorescence was assayed by flow cytometry on a BD Accuri C6 cytometer (Franklin Lakes, NJ, USA). Expression of APOBEC1 and AID was monitored by western blot. Cells were lysed (RIPA) and - after SDS-PAGE - the proteins were detected using either a primary goat anti-APOBEC1 antibody (1:5,000; Santa Cruz Biotechnology (Dallas, TX, USA), a monoclonal anti-AID antibody (hAnp52-1, 1:8,000) [64], or a beta-actin antibody (1:10,000; Sigma (St. Louis, MO, USA).

### Assaying APOBEC1 activity in DT40 cells

After electroporation with the APOBEC1/control constructs, DT40^GFP^ cells were plated in 96-well plates for selection. Independent DT40^GFP^ clones were used. Single clones were picked after approximately 8 days and expanded. We then analyzed 10^5^ cells by flow cytometry to assay the loss of EGFP fluorescence at 14 and 28 days after transfection. All experiments were repeated at least three times. For the mutation analysis, the GFP(−) population from independent clones was sorted using a BD FACSAria (Franklin Lakes, NJ, USA). After expansion the genomic DNA of the sorted population was prepared and the EGFP coding sequence was amplified by touch-down PCR using the KOD polymerase (CGTAAACGGCCACAAGTTCAG, ACTGGGTGCTCAGGTAGTGGT, 10 touchdown cycles plus 20 amplification cycles). The PCR fragments were cloned in pCR-Blunt II-TOPO according to the manufacturer’s instructions (Life Technologies, Carlsbad, CA, USA), and independent bacterial clones were sequenced (Additional file 9).

### Assaying APOBEC1 activity in K562 cells

Our failure to amplify by RT-PCR the catalytically active AID/APOBECs in K562 cells in the presence/absence of imatinib suggests that they are not expressed. This is in line with available data in GEO datasets GSE26821 and GSE51083. We have produced independent clones of K562 cells stably expressing APOBEC1-IRES-EGFP, AID-IRES-EGFP, or the control vector. We seeded 10^7^ cells from these clones in 96-well plates in medium supplemented with 1 μM of imatinib. Imatinib-resistant colonies grew after approximately 3 weeks, and were picked for expansion. After confirming the ability of these clones to grow in the presence of imatinib, total RNA was prepared and the mutations in the region encompassing exon 13 of *BCR* and exon 9 of *ABL1* were analyzed as described in [42]. The number of independent imatinib-resistant clones that were analyzed is: rat APOBEC1, 8; human APOBEC1, 5; AID, 6; control, 6. There are between 5 and 11 copies of the *BCR-ABL1* fusion gene in K562, with many of these copies inactivated by indels. In our sequencing we found evidence for at least five variants of the PCR-amplified *BCR-ABL1* fragment, only one of which could translate a proper BCR-ABL1 fusion protein. Typically - after cloning in a plasmid - we sequenced between 12 and 24 bacterial colonies for each imatinib-resistant clone in order to discriminate the active *BCR-ABL1* copy and obtain sufficient information on its mutational status (Additional files 10 and 11).

### Expression of APOBEC1 in the esophagus and mutational analysis

Expression data were downloaded from GEO [43]. The data from Kimchi *et al.* [45], Kim *et al*. [44], and Stairs *et al.* [46] correspond to GEO dataset accession numbers GSE1420, GSE13898 and GSE13083, respectively. All data were normalized on TATA-binding protein expression levels and their value centered on the median of the samples as Log2 values. Details of the statistics used are provided in the figure legends.

### Sequence context analysis

The sequence context of the mutations at cytosines in EACs was calculated using the mutation data from the exome analysis reported in Dulak *et al.* [47]. Only single-nucleotide changes were considered for analysis. Duplicate mutations were used only if originating from different tumor samples. For the analysis, we used the mutations at cytosines on both strands. Based on the genomic coordinates of the mutations, the local sequence context was extracted using a Perl script (Additional file 12). The expected mutational context was calculated based on the exonic sequences of the reference genome used for the exomes (build GRCh37). The trinucleotide representing the local context of mutations at C either from the DT40 experiments, from the EAC data, or from the control exome were then fed into the weblogo interface [48].

## Additional files

Additional file 1: Figure S1. Expression of the transduced proteins in stably transfected cells. **(A)** Western blot analysis of representative DT40 clones stably expressing APOBEC1. **(B,C)** Western blot analysis of representative K562 clones stably expressing rat APOBEC1, human APOBEC1, or AID as indicated. The blots were stripped and rehybridized with beta-actin antibody as control for loading. Additional file 2: Figure S2. Mutational sequence context in DT40 cells. Local sequence context for all residues present on both strands of the analyzed EGFP fragments (expected), for the mutated residues in the APOBEC1-expressing clones (APOBEC1) and in the controls (Control). Additional file 3: Figure S3. Expression levels of APOBEC1 in esophageal tissues. All data were normalized on TATA-binding protein (TBP) expression levels and shown as median-centered Log2 values. **(A)** Expression levels of the AID/APOBECs in the study by Kim *et al.* [44], comparing normal esophagus samples to esophageal adenocarcinoma (EAC) and Barrett’s esophagus samples. Each circle represents either a normal (blue), tumor (red), or Barrett’s (green) sample. The median is indicated as a black bar. Only differences in the expression levels of APOBEC1 (Normal/Tumor/Barrett’s samples) and APOBEC3A (Normal/Tumor samples) reach statistical significance (*P* < 0.0001 by one-way ANOVA coupled with Tukey’s test). **(B)** Levels of APOBEC1 in samples from matched normal/EAC pairs [44]. While an increased expression was present also with regards to APOBEC3A, only in the case of APOBEC1 did the differences reached statistical significance (*P* = 0.0002 by Wilcoxon signed rank test). **(C)** Levels of APOBEC1 in matched samples from normal esophagus, EACs, and Barrett’s esophagus in [45] (*P* = 0.0155 by repeated measures ANOVA coupled with Tukey’s test, with differences between tumors and Barrett’s samples not statistically significant). **(D)** Comparison of APOBEC1 expression levels in matched samples from normal/Barrett’s esophagus pairs and in small intestine [46] (*P* = 0.015 by Wilcoxon signed rank test). Additional file 4: Table S1. Possible cross-hybridization of the APOBEC3 probes in the Illumina GPL6102 chip. The data from Kim *et al.* [44] originate from the Illumina GPL6102 beadchip. This platform uses 50-mer probes that allow for a more specific analysis compared with the Affymetrix ones: some probes (A3A, A3C, A3D, A3F) could still cross-hybridize with other genes (albeit with non-transcribed/intronic portions of other APOBEC3s), and others (A3B, A3G, A3H) should not. The cross-hybridization potential of the individual probes (matching bases/length of the probe) is shown. The location of the probe on the gene if outside of the coding region is shown in parentheses (intron, in; untranscribed region in the 3’ of the gene, downstream). Additional file 5: Table S2. Mutational sequence context at position −2 in tumors expressing AID/APOBEC genes. The table shows the pyrimidine/purine ratio at position −2 in cancers for which the AID/APOBEC mutational signature has been associated with AID/APOBEC expression [30,31,33,58]. The expression of the various AID/APOBECs has been taken from Lin *et al.* [58] for the ESCC, and from Burns *et al.* [30,31] and Roberts *et al.* [33] for the other tumor types. The expression in EACs has been extrapolated from Kim *et al.* [44]. An asterisk indicates whenever the expression has been established through microarray analysis (as opposed to real-time PCR). The parentheses indicate the possible cross-reactivity of a microarray probe with other AID/APOBECs (Additional file 4). Additional file 6: Figure S4. Transition/transversion ratio in mutations at CpH sites in EAC associated or not with Barrett’s esophagus [47]. The number of tumors and the average in each subset are indicated in the legend and beside the box, respectively (*P* = 0.0192 by two-tailed *t*-test with Welch’s correction). The extra points represent the values external to the 5th to 95th percentile interval. Additional file 7: Figure S5. Sequence context for the mutated cytosine residues in EAC associated or not with Barrett’s esophagus [47]. The trinucleotide sequence context is shown for both CpG and non-CpG mutations as a weblogo [48]. Additional file 8:
**File containing the chromatograms of the sequences obtained from the Rifampicin assay.**
Additional file 9:
**File containing the chromatograms of the sequences obtained from the DT40 experiments.**
Additional file 10:
**File containing the chromatograms of the sequences obtained from the K562 experiments.**
Additional file 11:
**File containing the chromatograms of the sequences obtained from the K562 experiments.**
Additional file 12:
**File containing the Perl scripts used in the mutation analysis.**

## Abbreviations

AID: Activation Induced Deaminase
APOBEC1: Apolipoprotein B mRNA editing enzyme, catalytic polypeptide 1
bp: base pair
EAC: esophageal adenocarcinoma
EGFP: enhanced green fluorescent protein
FBS: fetal bovine serum
GEO: Gene Expression Omnibus
PCR: polymerase chain reaction
TBP: TATA-binding protein
