# Erratum: The RNA editing enzyme APOBEC1 induces somatic mutations and a compatible mutational signature is present in esophageal adenocarcinomas

**DOI:** 10.1186/s13059-014-0497-9

**Published:** 2014-11-08

**Authors:** Giulia Saraconi, Francesco Severi, Cesare Sala, Giorgio Mattiuz, Silvestro G Conticello

**Affiliations:** Core Research Laboratory, Istituto Toscano Tumori, viale Pieraccini 6, Firenze, 50139 Italy

## Erratum

During the typesetting of the final version of the article [1] some data in Table 1 have been accidentally changed. These data were correct in the provisional version of the article. A correct Table 1 is as follows:

**Table 1:** **List of the non-clonal mutations identified in the** ***BCR-ABL1*** **fusion gene from imatinib-resistant K562 clones**

**Samples**	**Position**	**AA change**	**Codon change**	**Sequence context**
Control	1409	E495G	G**A**A > G**G**A	
	1472	R491Q	C**G**G > C**A**G	GC**C**GG
	1503	Silent	TT**T** > TT**C**	
AID	298	G100C	**G**GC > **T**GC	GC**C**TA
	395	Silent	AA**A** > AA**G**	
	568	G190D	G**G**C > G**A**C	TG**C**CA
	633	T212A	**A**CG > **G**CG	
Rat APOBEC1	344	G155D	G**G**C > G**A**C	GG**C**CA
	607	L203M	**C**TG > **A**TG	CC**C**TG
	613	E205K	**G**AG > **A**AG	CT**C**GG
	764	K255T	A**A**G > A**C**G	
	987	Silent	GG**G** > GG**T**	TT**C**CC
Human APOBEC1	669	Silent	AG**C** > AG**T**	AG**C**CG
	758	R253H	C**G**C > C**A**C	TG**C**GC
	841*	Silent	**C**TG > **T**TG	AG**C**TG
	697	H233D	**C**AT > **G**AT	TT**C**AT
	699	H233D	CA**T** > GA**C**	
	1149*	Silent	GC**C** > GC**A**	GC**C**AT
	1245*	F415L	TT**T** > TT**G**	

The region analyzed (encompassing exon 13 of BCR and exon 9 of ABL1) includes the imatinib-binding region of the fusion gene. The asterisk indicates mutations found in the same clone. The local sequence context for the mutations at cytosines is shown. Compared to the AID-induced mutations found in previous reports (mutations in approximately 30% of the sequences) [41], [42], we found approximately one mutation in each of the clones analyzed. This is explained by the different procedures we used to select resistant clones: whereas the other studies focused on competing bulk populations of AID-transfected GFP(+) cells and control GFP(−) cells, we analyzed individual clones arising from the same number of cells plated in the presence of imatinib.

Please note that references 41 and 42 in the table legend correspond to the reference order in the original article [1].
