# Supplementary figures and images for: The RNA editing enzyme APOBEC1 induces somatic mutations and a compatible mutational signature is present in esophageal adenocarcinomas
Source: Genome Biol. 2014 Jul 31;15(7):417. doi: 10.1186/s13059-014-0417-z (PMC4144122; doi:10.1186/s13059-014-0417-z)

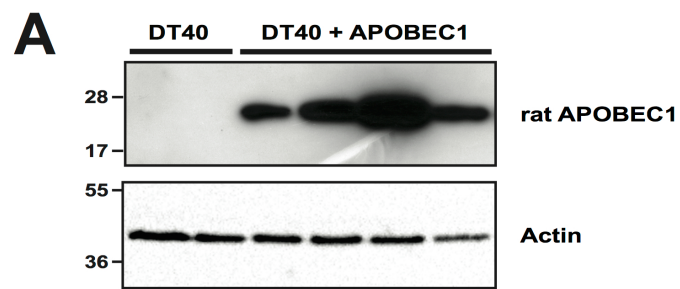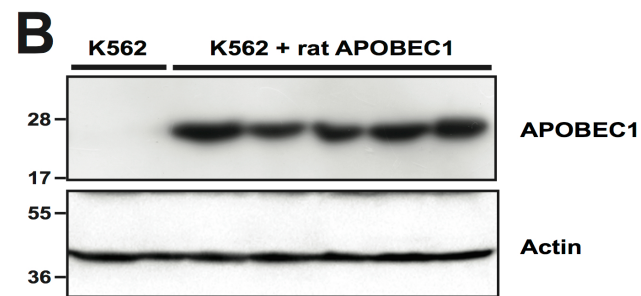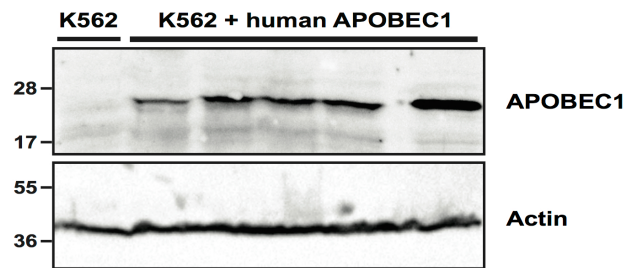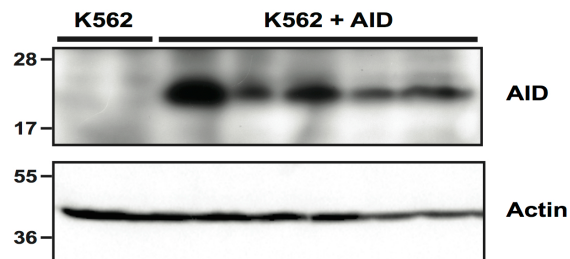

Supplement: Additional file 1: Figure S1. — Expression of the transduced proteins in stably transfected cells. (A) Western blot analysis of representative DT40 clones stably expressing APOBEC1. (B,C) Western blot analysis of representative K562 clones stably expressing rat APOBEC1, human APOBEC1, or AID as indicated. The blots were stripped and rehybridized with beta-actin antibody as control for loading. [file 13059_2014_417_MOESM1_ESM.pdf]

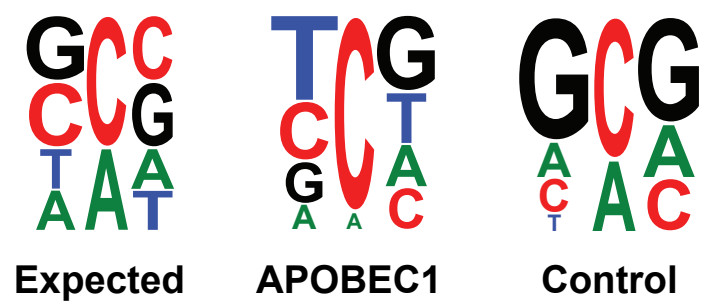

Supplement: Additional file 2: Figure S2. — Mutational sequence context in DT40 cells. Local sequence context for all residues present on both strands of the analyzed EGFP fragments (expected), for the mutated residues in the APOBEC1-expressing clones (APOBEC1) and in the controls (Control). [file 13059_2014_417_MOESM2_ESM.pdf]

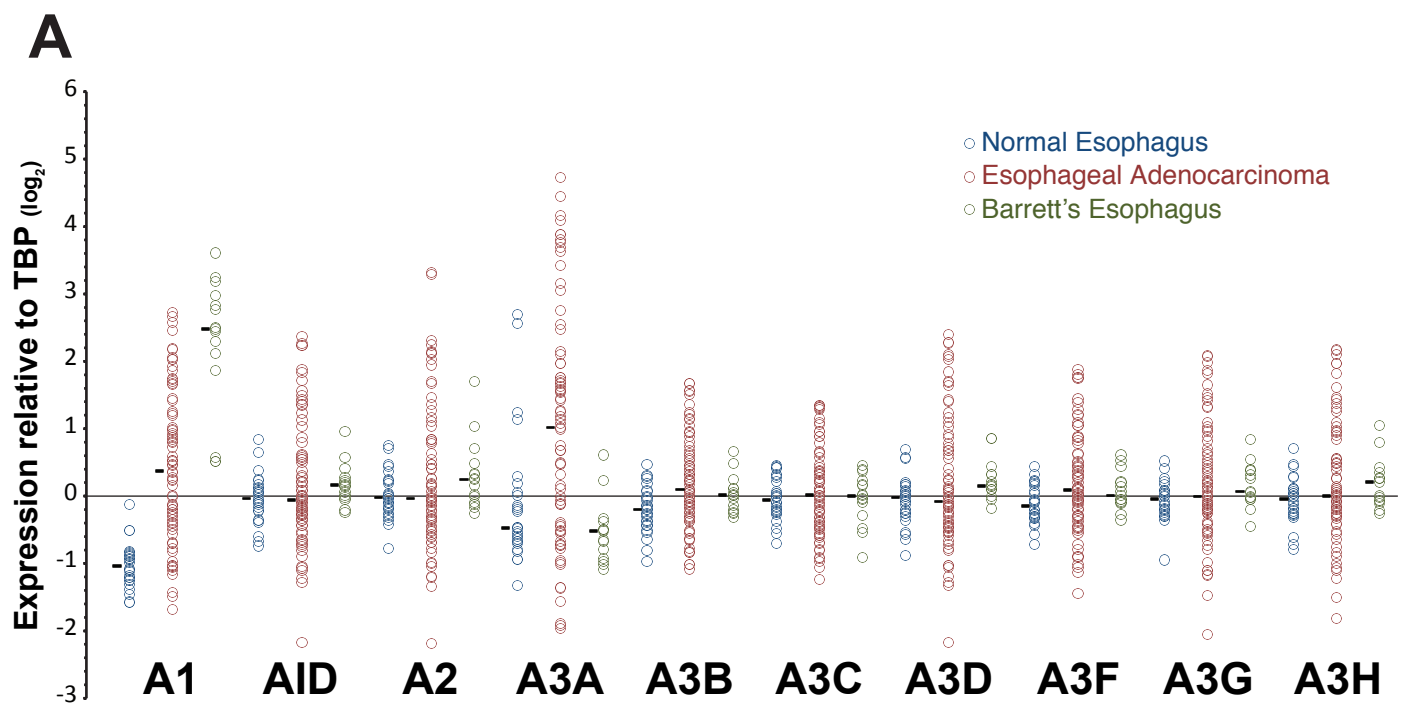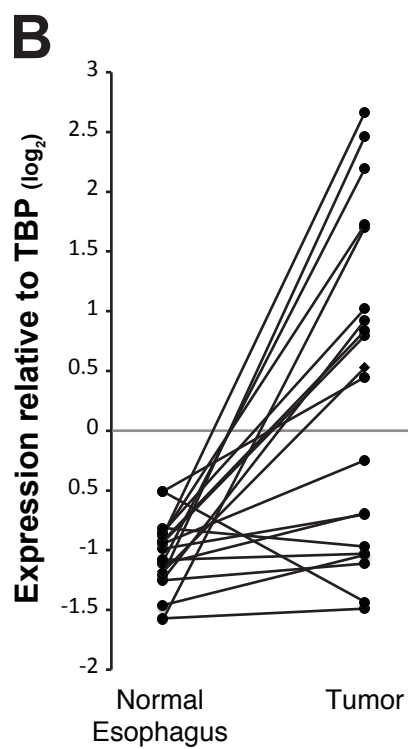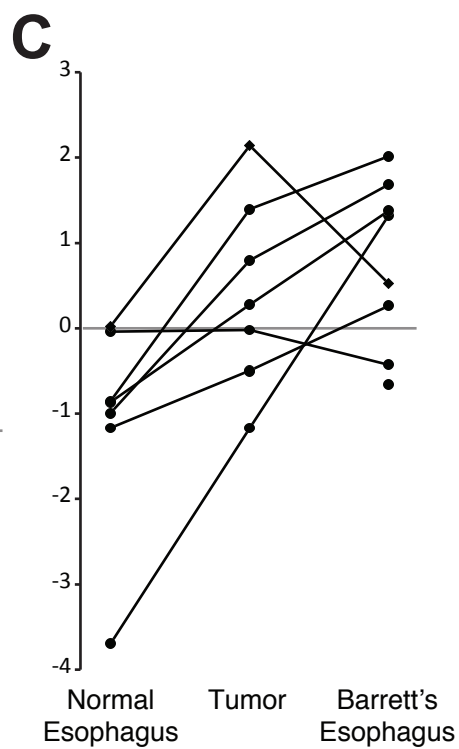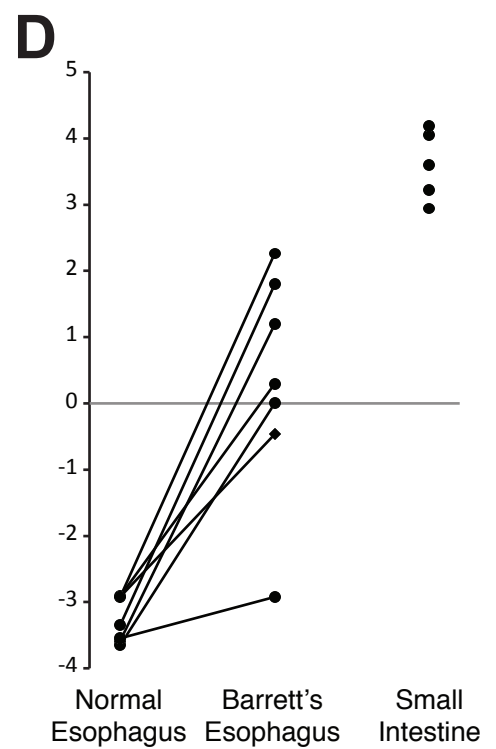

Supplement: Additional file 3: Figure S3. — Expression levels of APOBEC1 in esophageal tissues. All data were normalized on TATA-binding protein (TBP) expression levels and shown as median-centered Log2 values. (A) Expression levels of the AID/APOBECs in the study by Kim et al. [44], comparing normal esophagus samples to esophageal adenocarcinoma (EAC) and Barrett’s esophagus samples. Each circle represents either a normal (blue), tumor (red), or Barrett’s (green) sample. The median is indicated as a black bar. Only differences in the expression levels of APOBEC1 (Normal/Tumor/Barrett’s samples) and APOBEC3A (Normal/Tumor samples) reach statistical significance (P < 0.0001 by one-way ANOVA coupled with Tukey’s test). (B) Levels of APOBEC1 in samples from matched normal/EAC pairs [44]. While an increased expression was present also with regards to APOBEC3A, only in the case of APOBEC1 did the differences reached statistical significance (P = 0.0002 by Wilcoxon signed rank test). (C) Levels of APOBEC1 in matched samples from normal esophagus, EACs, and Barrett’s esophagus in [45] (P = 0.0155 by repeated measures ANOVA coupled with Tukey’s test, with differences between tumors and Barrett’s samples not statistically significant). (D) Comparison of APOBEC1 expression levels in matched samples from normal/Barrett’s esophagus pairs and in small intestine [46] (P = 0.015 by Wilcoxon signed rank test). [file 13059_2014_417_MOESM3_ESM.pdf]

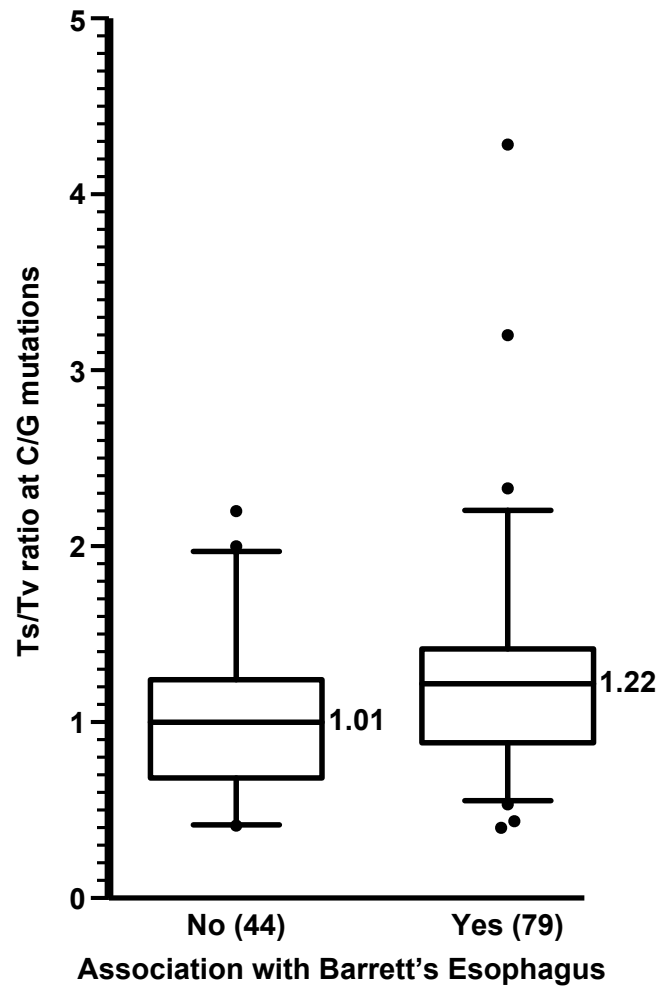

Supplement: Additional file 6: Figure S4. — Transition/transversion ratio in mutations at CpH sites in EAC associated or not with Barrett’s esophagus [47]. The number of tumors and the average in each subset are indicated in the legend and beside the box, respectively (P = 0.0192 by two-tailed t-test with Welch’s correction). The extra points represent the values external to the 5th to 95th percentile interval. [file 13059_2014_417_MOESM6_ESM.pdf]

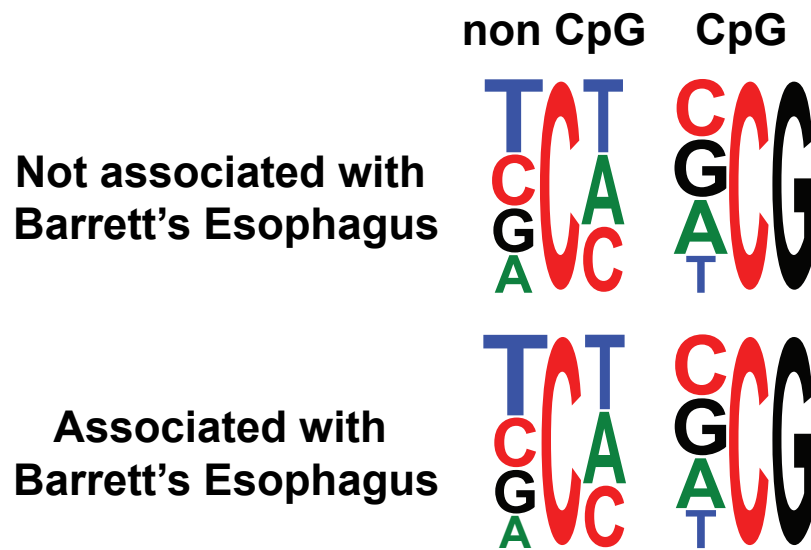

Supplement: Additional file 7: Figure S5. — Sequence context for the mutated cytosine residues in EAC associated or not with Barrett’s esophagus [47]. The trinucleotide sequence context is shown for both CpG and non-CpG mutations as a weblogo [48]. [file 13059_2014_417_MOESM7_ESM.pdf]
