# Supplementary material for: The RNA editing enzyme APOBEC1 induces somatic mutations and a compatible mutational signature is present in esophageal adenocarcinomas
Source: Genome Biol. 2014 Jul 31;15(7):417. doi: 10.1186/s13059-014-0417-z (PMC4144122; doi:10.1186/s13059-014-0417-z)
Supplement: Additional file 4: Table S1. — Possible cross-hybridization of the APOBEC3 probes in the Illumina GPL6102 chip. The data from Kim et al. [44] originate from the Illumina GPL6102 beadchip. This platform uses 50-mer probes that allow for a more specific analysis compared with the Affymetrix ones: some probes (A3A, A3C, A3D, A3F) could still cross-hybridize with other genes (albeit with non-transcribed/intronic portions of other APOBEC3s), and others (A3B, A3G, A3H) should not. The cross-hybridization potential of the individual probes (matching bases/length of the probe) is shown. The location of the probe on the gene if outside of the coding region is shown in parentheses (intron, in; untranscribed region in the 3’ of the gene, downstream). [file 13059_2014_417_MOESM4_ESM.pdf]

		AICDA	APOBEC1	APOBEC2	APOBEC3A	APOBEC3B	APOBEC3C	APOBEC3D	APOBEC3F	APOBEC3G	APOBEC3H
ILMN_1792434	**AICDA**	50/50									
ILMN_1813881	**APOBEC1**		50/50								
ILMN_1719143	**APOBEC2**			50/50							
ILMN_1680192	**APOBEC3A**				50/50					40/50 (downstream)	
ILMN_1691457	**APOBEC3B**					50/50					
ILMN_1675684	**APOBEC3C**						50/50	40/50	43/50		
ILMN_1661572	**APOBEC3D**					47/50 (in)		50/50	44/60		
ILMN_1710726	**APOBEC3F**						46/50	49/50	50/50		
ILMN_1698973	**APOBEC3F**						47/50 (in)	49/50 (in); 47/50 (in)	50/50; 44/50 (in)	49/50 (in); 48/50 (in)	
ILMN_1702706	**APOBEC3F**							50/50 (in)	50/50	50/50	
ILMN_1724169	**APOBEC3G**									50/50	
ILMN_1664828	**APOBEC3H**										50/50

Saraconi et al., Table S1
