# Supplementary material for: The RNA editing enzyme APOBEC1 induces somatic mutations and a compatible mutational signature is present in esophageal adenocarcinomas
Source: Genome Biol. 2014 Jul 31;15(7):417. doi: 10.1186/s13059-014-0417-z (PMC4144122; doi:10.1186/s13059-014-0417-z)
Supplement: Additional file 5: Table S2. — Mutational sequence context at position −2 in tumors expressing AID/APOBEC genes. The table shows the pyrimidine/purine ratio at position −2 in cancers for which the AID/APOBEC mutational signature has been associated with AID/APOBEC expression [30,31,33,58]. The expression of the various AID/APOBECs has been taken from Lin et al. [58] for the ESCC, and from Burns et al. [30,31] and Roberts et al. [33] for the other tumor types. The expression in EACs has been extrapolated from Kim et al. [44]. An asterisk indicates whenever the expression has been established through microarray analysis (as opposed to real-time PCR). The parentheses indicate the possible cross-reactivity of a microarray probe with other AID/APOBECs (Additional file 4). [file 13059_2014_417_MOESM5_ESM.pdf]

Tumor Type	AID/APOBEC expression	(TT+CT)/(AT+GT)	Number of tumors	*p*
Esophageal Adenocarcinoma (EAC)	A1*, (A3A*)	1.29 (0.08)	145	-
Lung Adenocarcinoma (LUAD)	A3B, A3D, A1	1.40 (0.55)	201	0.022
Breast Carcinoma (BRCA)	A3A, A3B, A3H, A1	1.52 (0.20)	969	0.01
Uterine Corpus Endometrioid Carcinoma (UCEC)	A3A, A3B	1.43 (0.45)	247	0.04
Lung Squamous Cell Carcinoma (LUSC)	A3B	1.50 (0.05)	176	0.61
Esophageal Squamous Cell Carcinoma (ESCC)	(A3B*)	1.63 (0.85)	124	0.001
Head and Neck Squamous Cell Carcinoma (HNSC)	A3B	1.75 (0.06)	508	0.001
Bladder Urothelial Carcinoma (BLCA)	A3B	2.37 (0.05)	237	10-16
Cervical Squamous Cell Carcinoma and endocervical adenocarcinoma (CESC)	A3B	2.44 (0.15)	40	10-7

Saraconi et al., Table S2
